# U1 snRNP Alteration and Neuronal Cell Cycle Reentry in Alzheimer Disease

**DOI:** 10.3389/fnagi.2018.00075

**Published:** 2018-03-23

**Authors:** Bing Bai

**Affiliations:** Department of Laboratory Medicine, Nanjing Drum Tower Hospital, Nanjing University Medical School, Nanjing, China

**Keywords:** Alzheimer’s disease, U1 snRNP, cytoplasmic redistribution, cell cycle reentry, inflammation

## Abstract

The aberrancy of U1 small nuclear ribonucleoprotein (snRNP) complex and RNA splicing has been demonstrated in Alzheimer’s disease (AD). Importantly, the U1 proteopathy is AD-specific, widespread and early-occurring, thus providing a very unique clue to the AD pathogenesis. The prominent feature of U1 histopathology is its nuclear depletion and redistribution in the neuronal cytoplasm. According to the preliminary data, the initial U1 cytoplasmic distribution pattern is similar to the subcellular translocation of the spliceosome in cells undergoing mitosis. This implies that the U1 mislocalization might reflect the neuronal cell cycle-reentry (CCR) which has been extensively evidenced in AD brains. The CCR phenomenon explains the major molecular and cellular events in AD brains, such as Tau and amyloid precursor protein (APP) phosphorylation, and the possible neuronal death through mitotic catastrophe (MC). Furthermore, the CCR might be mechanistically linked to inflammation, a critical factor in the AD etiology according to the genetic evidence. Therefore, the discovery of U1 aberrancy might strengthen the involvement of CCR in the AD neuronal degeneration.

## Introduction

Alzheimer’s disease (AD) is the most common dementia that is caused by the aging-related irreversible progression of neurodegeneration in the brain (Goedert and Spillantini, 2006; Roberson and Mucke, 2006). Besides the obvious brain atrophy with massive neuronal loss, AD brains are hallmarked by the deposition of extracellular amyloid plaques and intracellular neurofibrillary tangles, whose major components are Aβ peptides and the hyperphosphorylated microtubule-associated protein Tau (MAPT), respectively.

Currently, all three established familial AD genes (APP, PSEN1 and PSEN2) are directly involved in the Aβ generation (Guerreiro et al., 2013). Mutations that lead to enhanced Aβ production or fibrillization accelerate the onset of AD (Citron et al., 1992; Nilsberth et al., 2001), and those inhibiting Aβ generation reduce the likelihood of AD development (Jonsson et al., 2012). In combination with other lines of evidence from pathology (Serrano-Pozo et al., 2011; Selkoe, 2013), biochemistry (Cleary et al., 2005; Masters and Selkoe, 2012), cell biology (Shankar et al., 2008; Kuperstein et al., 2010) and animal models (Lesne et al., 2006; Oakley et al., 2006), it is well accepted that the amyloid cascade is the initiating event in the AD pathogenesis (Selkoe et al., 2012; Bloom, 2014; Selkoe and Hardy, 2016). However, the brain Aβ load has a fair correlation with the severity of dementia in patients (Giannakopoulos et al., 2003; Nelson et al., 2012); and the Aβ overexpression in mouse brains fails to elicit the dementia and neurofibrillary tangles to the extent manifested in AD patients (Oakley et al., 2006).

Although the neurofibrillary tangles have a better correlation with the severity of dementia, they are not specific for AD and appear in almost any kinds of brain diseases (Nelson et al., 2012). In addition, the neuronal stress has already been observed prior to tangle formation in the neuron and the number of dying neurons is often greater than the tangle-bearing neurons (Gómez-Isla et al., 1997; Hoozemans et al., 2009). Moreover, almost all people will eventually develop tangles during aging even their cognition is intact (Nelson et al., 2012). Collectively, the tangle formation might possibly be considered as a universal event that appears at the later stage of the neuronal dyshomeostasis. Therefore, the critical pathway besides the amyloid cascade that mediates the final neuronal degeneration in the AD pathogenesis still remains unclear.

## U1 snRNP Proteopathy and RNA Splicing Deficiency in AD

It is known that some neurodegenerative disorders are caused by the aberrancy of RNA processing proteins (Neumann et al., 2006; Sreedharan et al., 2008; Mackenzie et al., 2010), but whether the pathogenesis of AD possibly involves a similar mechanism had remained unclear, until we have found the U1 small nuclear ribonucleoprotein (snRNP) complex pathology and the RNA splicing deficiency in AD patents (Bai et al., 2013).

The major human spliceosome is composed of five subunits, each comprising snRNPs in conjunction with a specific small nuclear RNA (snRNA) named as U1, U2, U4, U5 and U6 respectively (Wahl et al., 2009). The U1 snRNP contains the U1 snRNA in complex with U1-70K, U1A, SmD and other protein components, which are normally located in the neuronal nucleus. However, these proteins are aggregated and form tangle-like structures in the neuronal cytoplasm in AD brains (Bai et al., 2013; Hales et al., 2014a).

Notably, this U1 proteopathy, unlike Tau, is almost exclusively present in AD and Down’s syndrome (Bai et al., 2013; Hales et al., 2014b), but not in any other types of dementia or neurodegenerative diseases that involve no amyloid and Tau tangle pathologies, providing a unique clue to the mechanism of AD. Importantly, in these reports, the U1 aggregation occurs in the mild cognitive impairment, an early stage of dementia and often evolves into AD eventually.

Besides, we have also found that the RNA splicing is impaired in the AD brains, probably as a functional consequence of the U1 proteopathy. The transcriptomic analysis by RNA deep sequencing shows that the overall intronic reads are increased in AD as compared to those in the non-demented controls. Further examination reveals that the insufficient RNA splicing occurs extensively in individual genes. Nevertheless, the strongest evidence of U1 dysfunction might come from the phenomenon of PCPA (premature cleavage and polyadenylation) observed in the RNA transcriptome data. Besides RNA splicing, the U1 SnRNP complex has a unique function. It prevents the PCPA of the pre-RNA during transcription through binding to the putative polyadenylation sites (Kaida et al., 2010). This is also found the AD brains in our study in addition to the evidence of RNA splicing deficiency (Bai et al., 2013), confirming the dysfunction of U1 proteopathy. The following up studies by other groups demonstrate more possible roles of U1 in the autophagy-lysosome system and the presenilin protein (Cheng et al., 2017a,b, 2018), both of which are key players in the AD etiology. Indeed, the U1 deregulation in AD on particular genes has been reported previously (Manabe et al., 2007; Ohe and Mayeda, 2010).

## Spliceosome Alterations Cause Neurodegeneration

It is well known that alterations in the RNA processing machineries can cause neurodegeneration. The first example is the spinal muscular atrophy (SMA), arising from mutations in the gene SMN that encodes the survival motor neuron protein (Lefebvre et al., 1995, 1997; Lorson et al., 1999). The SMA disease is characterized by the loss of motor neurons and the progressive muscle atrophy. The SMN protein, together with other proteins, forms the heptameric protein ring that commonly exists as a core in the U1, U2, U4, U5 and U6 snRNP complexes to catalyze their assembly (Matera and Wang, 2014; Wahl and Lührmann, 2015). The homozygous disruption of SMN causes this inheritable neuromuscular disorder and often leads to death in patients (Lunn and Wang, 2008). Consistently, experimental reduction in the SMN level in zebra fish or mice leads to motor neuron degeneration and its restoration rescues this deleterious effect (Winkler et al., 2005; Hua et al., 2011).

The next strong evidence is about U2 snRNA, the specific component of U2 snRNP subunit. Mutations in one of the copies of U2 snRNA genes lead to ataxia in mice, with extensive neurodegeneration and RNA splicing aberrancy in the cerebellum where the U2 snRNA is highly expressed (Jia et al., 2012). Another example is from hnRNPA2B1 and hnRNPA1, both of which are involved in the RNA processing and splicing regulation (Gabut et al., 2008). Their mutations have been found in the familial multisystem proteinopathy and ALS, two devastating diseases resulting from the progressive degeneration of the neural system; and expression of the mutant hnRNPA2B1 and hnRNPA1 in transgenic *Drosophila* recapitulates the phenotype and pathology to some extent demonstrated in human (Kim et al., 2013). These lines of evidence support the notion that disruption in the RNA splicing system is sufficient to cause the degeneration of the neural system.

The protein TDP-43 binds DNA and RNA to regulate the transcription and splicing processes. Although it is not a typical member of the spliceosomal proteins based on the currently available knowledge (Hegele et al., 2012; Korneta et al., 2012), proteopathy of TDP-43 was discovered in the central nervous system that includes hippocampus, neocortex, and spinal cord in patients with frontotemporal lobar degeneration (FTLD-U) or amyotrophic lateral sclerosis (ALS; Neumann et al., 2006; Maekawa et al., 2009). Further genetic evidence establishes the causative role of TDP-43 in these two neurodegenerative diseases by its mutations in the familial cases (Sreedharan et al., 2008), strengthened by evidence from other biological studies (Gitcho et al., 2008; Wegorzewska et al., 2009; Wils et al., 2010; Alami et al., 2014).

Despite the direct evidence of mutations in U1 snRNP components is currently rare, probably because their knockouts are embryonically lethal (Hilleren et al., 1995; Salz et al., 2004); and the direct association between the U1 proteopathy and the cellular stress within the same neuron in the brain is currently under investigation, the U1 dysfunction in AD brains is presumably a disaster in neurons. However, what causes it? Insights might be gained from the characteristics of its histopathology in the brain.

## Cytplasmic Distribution of U1 snRNP in AD Brain Neurons

The prominent feature of the neuronal U1 pathology in AD brains is its depletion from the nucleus and redistribution in the cytoplasm where it largely overlays with the neurofibrillary phospho-Tau (Bai et al., 2013). Besides, when the phospho-Tau is not obvious yet in the neuron, we often find U1-70K has already redistributed into the cytoplasm surrounding the nucleus without forming the tangle-like structure (Data to be published). This not only indicates that the U1 alteration and the Tau proteopathy are two independent events, but also suggests that the original characteristic of the U1 pathology is its nuclear exclusion redistribution into the cytoplasm.

Actually, the cytoplasmic redistribution of nuclear proteins is quite common in neurodegenerative disorders. TDP-43 and FUS, the nuclear proteins that bind RNA/DNA, are normally located in the neuronal nucleus, but relocate into the cytoplasm and colocalize with the ubiquitin-positive inclusion body in brains or spines of FTLD-U and ALS patients (Arai et al., 2006; Neumann et al., 2006). SFPQ, another nuclear RNA/DNA binding protein that is mainly involved in RNA splicing, relocates from the nucleus to the perinuclear region of the cytoplasm in the hippocampal neurons in AD and Pick’s Disease (Ke et al., 2012). In addition, similar nuclear exclusion phenomenon is also seen with the proteins hnRNPA2/B1 which form aberrant sarcoplasmic inclusions in multisystem proteinopathy and ALS patients, and also in the animal models (Kim et al., 2013). However, unlike TDP-43 and FUS whose cytoplasmic accumulations appear focal and might be related to stress granules, the U1 snRNPs show a more diffusive distribution pattern and display filamentous structure (Hales et al., 2014b), indicating a distinct underlying mechanism.

## U1 snRNP Cytoplasmic Redistribution Indicates Neuronal Cell Cycle Reentry (CCR)

The first possible biological event that causes the U1 subcellular location change is the apoptosis (Dieker et al., 2008). During this cellular process, U1-70K is phosphorylated, fragmented, and largely excluded from the DNA-staining region and become surrounding the chromatins in clusters. However, it is notable that this cluster-like appearance is largely different from that observed in the AD brain, in which the distribution of U1 is more diffusive (Bai et al., 2013). This is consistent with the fact that apoptosis is not likely the major way of neuronal death in AD (Stadelmann et al., 1998; Yuan and Yankner, 2000; Zhu et al., 2006). Indeed, the U1-70K fragment in the AD brain is not generated through cleavage by caspase-3 (Bai et al., 2014). Therefore, the U1 subcellular mislocation is not likely a reflection of apoptosis.

The next event that elicits the U1 subcellular distribution in a pattern similarly observed in AD is mitosis. In this cellular process, the U snRNPs initially remain in the nucleus in the interphase, then move to the cytoplasm after the nuclear envelope is broken down during the metaphase and anaphase, and finally return into the nuclei of the two daughter cells in the telophase (Verheijen et al., 1986). The nucleus-to-cytoplasm redistribution of spliceosome during mitosis is extensively demonstrated in many studies (Goldstein et al., 1977; Reuter et al., 1985; Spector and Smith, 1986; Leser et al., 1989; Carmo-Fonseca et al., 1993; Azum-Gélade et al., 1994; Ferreira et al., 1994; Blencowe, 2003). It is worth reiterating that the cytoplasmic distribution of U1 snRNPs during the mitosis is very similar to the U1 pathology when Tau tangles are not present in AD brains (Supplementary Figure S1).

Indeed, the neuronal cell cycle activation has already been widely evidenced. Neurons are postmitotic and usually not dividable, in which the cell cycle is arrested in the G1 phase. However, a significant number of hippocampal pyramidal and basal forebrain neurons in AD brains display duplicated genetic loci on different chromosomes (Yang et al., 2001), indicating a progression from G1 phase into the S phase. In addition, several critical cell proliferation and cycle-related proteins, such as PCNA (proliferating cell nuclear antigen), cyclin D and cyclin B, are evidenced to increase in hippocampus, basal nucleus of Meynert, and entorhinal cortex in AD brain sections (Yang et al., 2003). Similar cell cycle-reentry (CCR events have been extensively demonstrated and studied by several major research groups (Andorfer et al., 2005; Bauer and Patterson, 2005; Webber et al., 2005; Neve and Mcphie, 2006; Herrup and Yang, 2007; Varvel et al., 2008). The major CCR related molecules and events in AD human and animals are summarized (Table 1).

**Table 1:** Cell cycle related molecules and events in Alzheimer’s disease (AD).

Molecules/insults	Description
Expression of cell cycle related events or proteins in AD	
DNA replication	Fully or partially replicated separate genetic loci on some chromosomes identified by fluorescent *in situ* hybridization (Yang et al., 2001).
PCNA	Increased expression in the hippocampus and other regions in AD brains (Busser et al., 1998; Yang et al., 2003).
Cyclin B	Increased expression in certain regions in AD. Neurons with high expression of cyclin B has phosphorylated Tau, but not necessarily the tangle-like Tau (Nagy et al., 1997; Smith et al., 1999; Yang et al., 2003).
Cyclin D	Increased expression in the hippocampus and other regions in AD brains (Busser et al., 1998; Yang et al., 2003).
Cyclin E	It is also expressed more in AD brains (Nagy et al., 1997; Smith et al., 1999).
CDK4	Increased expression in AD brains (McShea et al., 1997).
P16 (CDKN2A)	Increased expression in AD brains (McShea et al., 1997).
CARB	Associated with p21 and cyclin B, involved in cell cycle; colocalizes with the tangle and granulovacuolar degeneration in AD brain neurons (Zhu et al., 2004).
c-myc and ras	Drives DNA replication and expression of cyclin B in cultured primary cortical neurons, also induces phosphorylation and conformational change of Tau (McShea et al., 2007).
p38 MAPK	Diffusively distributed in the cytoplasm in the controls, while completely overlapped with Tau tangle in AD brains in the hippocampus and cortex (Zhu et al., 2000).
RGCC	Increases in MCI and AD; and correlates with the cognitive deficit (Counts and Mufson, 2017).
BRCA1	Colocalization with the neurofibrillary tangles (Evans et al., 2007).
Mcm2	Involved in DNA replication and becomes phosphorylated by CDKs and Cdc7 during DNA synthesis (Bonda et al., 2009).
Linkage between the cell cycle and the Alzheimer proteins	
APP	Phosphorylated at Thr668 in AD; phosphorylation of this site occurs during cell cycle by cdc2 kinase; the APP-binding protein (APP-BP1) is also able to trigger cell cycle progression through NEDD8 pathway (Suzuki et al., 1994; Chen et al., 2000).
Aβ	Aβ oligomers induces CCE in cultured primary neurons via Tau (Seward et al., 2013); neuronal CCE prior to Aβ deposition occurs in the APP transgenic rat brains (Varvel et al., 2008).
Tau	It can induce cell cycle related proteins and DNA synthesis in transgenic mice that overexpress human Tau (Andorfer et al., 2005; Hradek et al., 2015).
Presenilin	Overexpression arrests the cell cycle in the G1 Phase; the AD mutant promotes cell cycle arrest; presenilin deficiency in mice delays the cell cycle (Janicki and Monteiro, 1999; Janicki et al., 2000; Yuasa et al., 2002).
Cell cycle triggering or regulatory molecules and events	
TNF-α	Microglial-derived TNFα induces neuronal CCE via the JNK signaling; microglia extracted from the APP transgenic mice (R1.40) drives neuronal CCEs in the host mouse brain and this can be blocked by Tnfα knockout (Bhaskar et al., 2014).
Oxidative stress	Induce CCE via DNA damage or other mechanisms (Klein and Ackerman, 2003; Lin and Beal, 2006; Silva et al., 2014).
AGEs	Indicator of oxidative stress; increased level in AD brain; colocalizes with neurons expressing cyclin D and DNA replications signs (Kuhla et al., 2015).
DNA Damage	It induces cell cycle reentry in cultured primary postmitotic neurons (Kruman et al., 2004).
Cerebral ischemia	Transient Cerebral Ischemia induces expression of mitotic proteins and tau phosphorylation in adult female rat cortex (Wen et al., 2004). Mild Cerebral Ischemia Induces Loss of CDKN2A and activation of CCE to neuronal death (Katchanov et al., 2001).
Hypoxia-Ischemia	Induces increased expression of Ki67, reduced p16INK4 and p27Kip1, upregulated CDK2 activity, and phosphorylation of Rb (Kuan et al., 2004).
Excitotoxicity	Kainic-acid treatment *in vivo* induces erroneous CCR in cultured primary postmitotic neurons through the Notch signaling (Marathe et al., 2015).
MiR-26b	Increased expression in AD brains as early as at Braak III; triggers DNA replication and CCE, tau phosphorylation in cultured neurons (Absalon et al., 2013).

## Neuronal CCR Explains Major Cellular and Molecular Alterations in AD

The neuronal CCR theory gains more research attention because it explains several critical events during the AD pathogenesis. The first is the Tau phosphorylation, one of the hallmarks in AD pathology. As a member of the Ser/Thr cyclin-dependent kinases, CDK5 phosphorylates Tau at the sites that are most frequently hyperphosphorylated in AD brains (Kimura et al., 2014). CDK5 is active mainly in the postmitotic neurons where its regulatory protein subunits p35/p39 are predominantly expressed and activated in the AD brain (Patrick et al., 1999). Actually, the fact of Tau phosphorylation during mitosis is evidenced by the *in vitro* study (Illenberger et al., 1998). Therefore, it is reasonable to speculate that the Tau hyperphosphorylation possibly involves the CCR attempt in AD brain neurons.

The neuronal CCR might also account for the APP phosphorylation in AD brains. The phosphorylation of T668 on APP695, the major form in neurons (Kang and Müller-Hill, 1990), is known to be significantly increased in AD and leads to accelerated Aβ generation. Further study has demonstrated that the phosphorylation of APP on T668 can be achieved during cell cycle by CDK5 and CDC2 kinases (Suzuki et al., 1994; Iijima et al., 2000; Liu et al., 2003). As a critical evidence, such phosphorylated APP is largely accumulated in neurons that bear phosphorylated Tau (Lee et al., 2003), strongly suggestive of CCR as their common upstream inducer.

Besides, the neuronal CCR might provide insights to the mechanism of AD neuronal death. As mentioned earlier, the AD neuronal loss is not likely due to apoptosis. If CCR is widely activated, then the mitotic catastrophe (MC) might be a mechanism of neuronal death in AD. The MC is a type of cell death that results from failed completion of mitosis (Kroemer et al., 2009). In AD hippocampus, the phosphorylated histone H3 appears in the neuronal cytoplasm instead of its normal localization in the nucleus during mitosis in actively dividing cells, indicating an aberrant mitosis undergoing in neurons which might proceed into necrosis by MC (Ogawa et al., 2003). Okadaic acid (OA), a potent phosphatase inhibitor, induces the expression of G2/M phase cyclins B1 and D1 in neuroblastoma cells to activate cell cycle, making neurons become dying with signs of MC (Chen et al., 2006). Interestingly, these OA treatments can induce the paired helical filament-like phosphorylation of Tau in rats (Arendt et al., 1995), possibly through the activation of CDK5. Collectively, these lines of evidence suggest CCR can be activated in neurons and lead to MC eventually, providing a potential mechanism of neuronal death in AD.

Indeed, the aging process is associated with the activation of cell cycle and this is well demonstrated in the mouse model. The senescence-accelerated mice 8 (SAMP8) is a model of aging and displays typical AD pathological characteristics (Pallas et al., 2008), including Aβ amyloid accumulation and Tau phosphorylation and other events (Del Valle et al., 2010). The SAMP8 mice not only have elevated CDK5 and GSK3β, but also demonstrate a significant increase of cell cycle progression markers, including cyclin A, cyclin D1, cyclin E, Cdk2, cyclin B, pRb, and E2F1 (Casadesüs et al., 2012). Taken together, lessons from the SAMP8 mice might highlight the CCR during the aging process as a major driver in the AD pathogenesis.

## Mechanistic Link Between Neuronal CCR and Inflammation in AD

Study has shown the soluble Aβ oligomers induce neuronal CCR through the phosphorylation of Tau (Seward et al., 2013). The CCR events are also observed in APP transgenic mice at about ~6 months of age at which a substantial amount of soluble Aβ peptides are expressed (Varvel et al., 2008). However, the CCR events are usually only sparsely seen in these animal models. The more extensive CCRs in AD brains likely involve other factors.

Inflammation is probably the most important causative insult besides the amyloid cascade in the AD pathogenesis (Wyss-Coray and Rogers, 2012; Heppner et al., 2015); and it is able to trigger the cell cycle process. Because the activation of cell cycle is closely related to the cellular proliferation which is a hallmark in tumorigenesis, insights about the role of inflammation in AD neuronal CCR might be gained from its role as a driver in the cancer development (Crusz and Balkwill, 2015).

The cancer and AD are apparently two opposite diseases with a common molecular basis: loss of control on cellular growth due to chronic accumulation of biological alterations (López-Otín et al., 2013). Therefore, the neuronal CCR might be considered as a result of an aborted tumorigenesis. In fact, both diseases have age as their strongest risk factor; and presents a similar incidence trend during aging: from ~4% under 65 years old and up to 40% after 75 years old (Crusz and Balkwill, 2015; Siegel et al., 2018). Because these two diseases are probably the opposite manifestations of the same disorder, they tend to be exclusive in a particular individual and therefore have a negative association in a general population as expected (Roe et al., 2010; Musicco et al., 2013).

The inflammation drives the cancer development through factors including IL-1, IL-6, IL-13, IL-22, TNFα, TGFβ, ROS and other possible agents, with a converge on two major signaling pathways: STAT3 and NF-κB (Elinav et al., 2013). The IL-1 and TNFα activate the NF-κB pathway to increase the expression of IL-6 which, in turn, stimulates through STAT3 the upregulation of cyclins D1, D2 and B to initiate the cell cycle for cellular proliferation. This pathway is widely demonstrated in cancer tissues where it associates with the inflammation (He and Karin, 2011; Dmitrieva et al., 2016; Taniguchi and Karin, 2018), suggesting a causative relationship between the inflammation and the cell cycle activation. Among these factors, IL-1, IL-6 and TGF-β are evidenced to be increased in AD brains (Blum-Degen et al., 1995; Alvarez et al., 1996; Ye and Johnson, 1999; Luterman et al., 2000; Quintanilla et al., 2004; Patel et al., 2005; Rota et al., 2006; Ghosh et al., 2013; Zheng et al., 2016), in which STAT3 and NF-κB pathways are also activated (Tarkowski et al., 2002; Tesseur et al., 2006; Town et al., 2008; Chiba et al., 2009; Wan et al., 2010; Ben Haim et al., 2015). Besides, the complements of the innate immune system that are upregulated and activated in AD, can also modulate the cell cycle (Rus et al., 1996, 2001; Fosbrink et al., 2005). Therefore, the inflammation in AD brains, probably initiated from amyloid plaques but exacerbated by other possible factors (e.g., chronic accumulation of other aberrant proteins, the blood-brain barrier leakage, the reactivation of latent microorganisms, etc.; Glass et al., 2010), might be the major inducer for the fatal CCR in neurons.

## Conclusion

**Figure 1:**
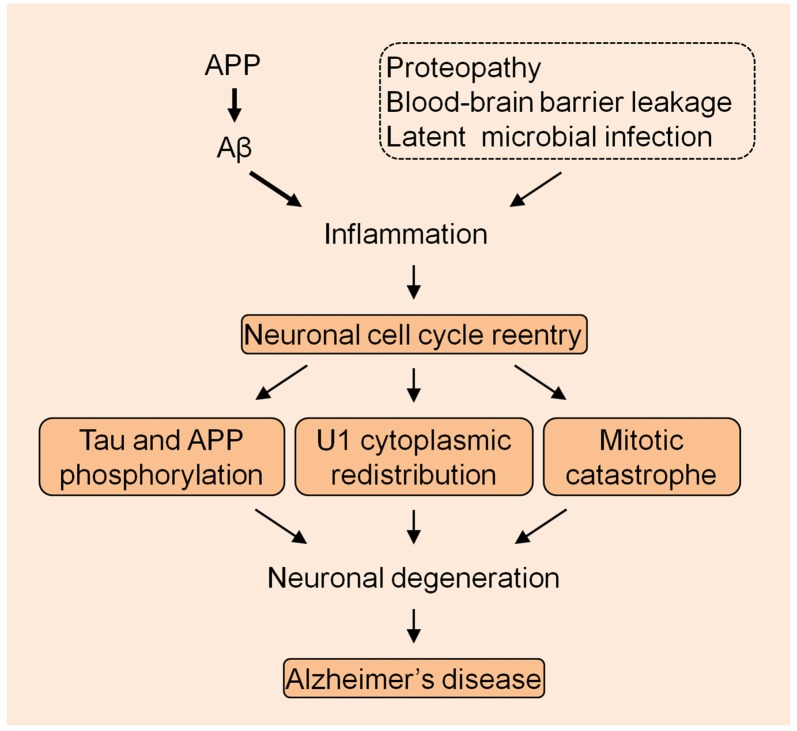
The summarized model of Alzheimer’s disease (AD) pathogenesis. The amyloid precursor protein (APP) derived Aβ species initiates the inflammation with the exacerbation by other insults to drive the neuronal cell cycle events.

The U1 snRNP pathology provides a very unique mechanism in the AD pathogenesis. Although other mechanisms might exist, the most possible cellular alteration that mechanistically links U1 alteration is the neuronal cell cycle reentry, based on the preliminary data that we have obtained. It is possible that AD is caused by the continued excessive accumulation of Aβ that elicits the immune response which is exacerbated by other inflammatory insults, initiating the neuronal cell cycle activation that eventually causes neuronal death by MC (Figure 1). Nevertheless, thorough studies are required to evaluate this bold assumption, which will facilitate understanding of the fundamental mechanism of the AD etiology.

## Author Contributions

BB conceived the idea and wrote the manuscript.

## Conflict of Interest Statement

The author declares that the research was conducted in the absence of any commercial or financial relationships that could be construed as a potential conflict of interest.

## Supplementary Material

FIGURE S1 Immunofluorescent staining to demonstrate the cytoplasmic distribution of U1-70K in a Alzheimer’s disease (AD) brain and HEK293T cells. **(A)** Coimmunostaining of U1-70K and phospho-Tau (AT8) on an AD brain cortical tissue slide. The dotted circles point to the neuron with nuclear depletion and cytoplasmic distribution of U1-70K, but no obvious phospho-Tau staining. **(B)** U1-70K in HEK293T cells. The dotted circles point the cytoplasmic distribution of U1-70K in cells that seem in the mitotic process. DAPI: 4′,6-Diamidine-2′-phenylindole dihydrochloride, a DNA staining dye.

## Abbreviations

AD: Alzheimer’s Disease
APP: amyloid precursor protein
CCR: cell cycle reentry
MC: mitotic catastrophe
snRNP: small nuclear ribonucleoprotein.
