# Supplementary figures and images for: U1 snRNP Alteration and Neuronal Cell Cycle Reentry in Alzheimer Disease
Source: Front Aging Neurosci. 2018 Mar 23;10:75. doi: 10.3389/fnagi.2018.00075 (PMC5876301; doi:10.3389/fnagi.2018.00075)

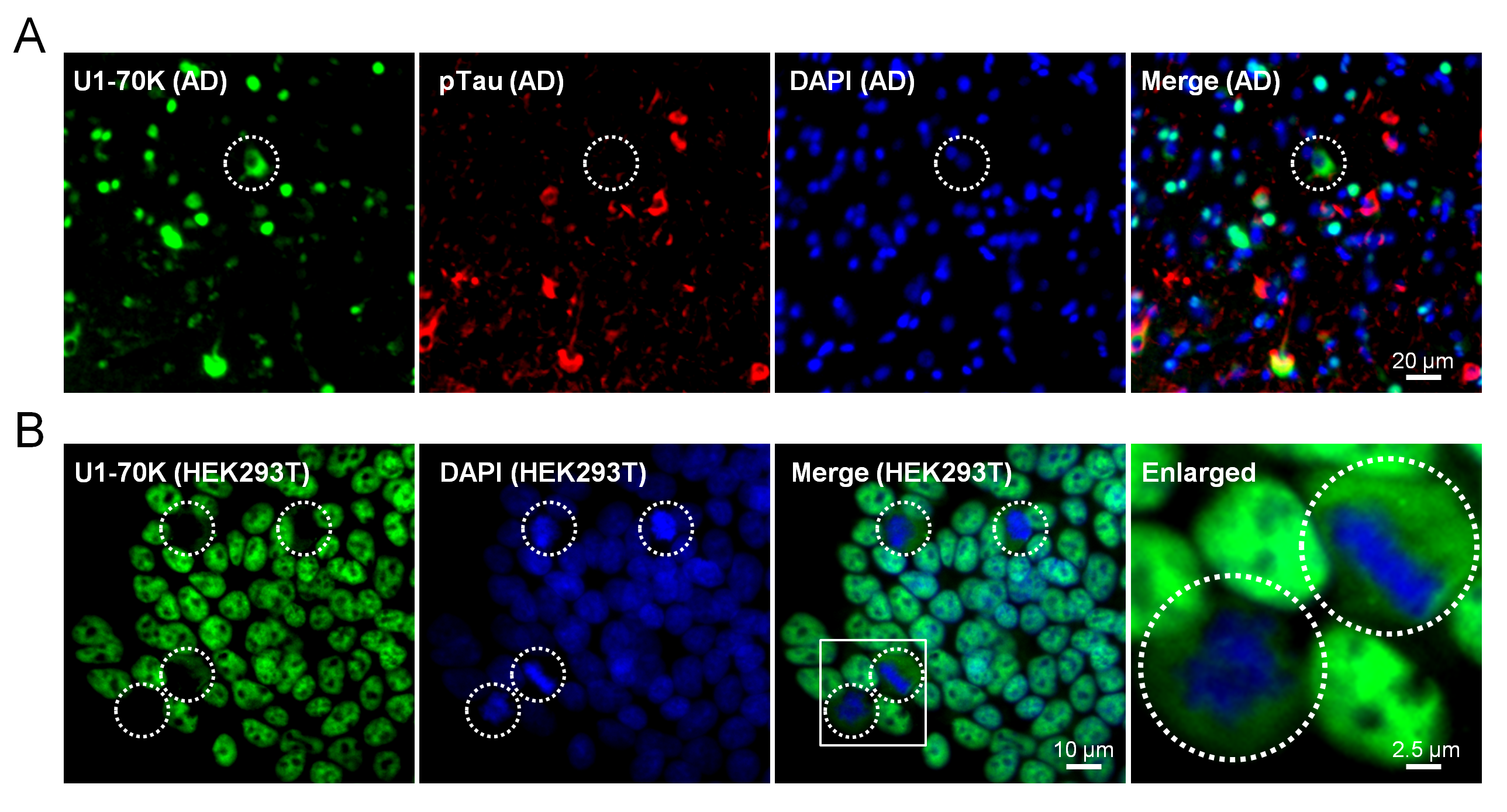

Supplement: FIGURE S1 — Immunofluorescent staining to demonstrate the cytoplasmic distribution of U1-70K in a Alzheimer’s disease (AD) brain and HEK293T cells. (A) Coimmunostaining of U1-70K and phospho-Tau (AT8) on an AD brain cortical tissue slide. The dotted circles point to the neuron with nuclear depletion and cytoplasmic distribution of U1-70K, but no obvious phospho-Tau staining. (B) U1-70K in HEK293T cells. The dotted circles point the cytoplasmic distribution of U1-70K in cells that seem in the mitotic process. DAPI: 4′,6-Diamidine-2′-phenylindole dihydrochloride, a DNA staining dye. [file Image_1.TIF]
